# Pinch-off syndrome leading to catheter fracture: a rare complication of central venous port systems, a case report

**DOI:** 10.1093/bjrcr/uaaf056

**Published:** 2025-12-27

**Authors:** Soumya El Graini, Ibtissam El Ouali, Hamza Retal, Youssef Omor, Rachida Latib, Sanae Amalik

**Affiliations:** Radiology Department, National Institute of Oncology, Rabat 6527, Morocco; Radiology Department, National Institute of Oncology, Rabat 6527, Morocco; Radiology Department, National Institute of Oncology, Rabat 6527, Morocco; Radiology Department, National Institute of Oncology, Rabat 6527, Morocco; Radiology Department, National Institute of Oncology, Rabat 6527, Morocco; Radiology Department, National Institute of Oncology, Rabat 6527, Morocco

**Keywords:** pinch-off syndrome, catheter migration, central venous port systems

## Abstract

Totally implantable venous access devices provide long-term venous access for patients requiring extended therapies, such as chemotherapy, with lower risks of infection and extravasation. These devices are typically placed in the subclavian or internal jugular vein and connected to a subcutaneous port. Following implantation, a chest X-ray is used to confirm catheter placement, detect early complications, and identify the “pinch-off sign”—a warning of potential catheter compression between the clavicle and first rib. Complications include malposition, pneumothorax, infection and catheter dysfunction such as pinch-off syndrome (POS), catheter fracture, and migration. POS can cause catheter dysfunction, fracture, and migration, potentially leading to severe cardiovascular or neurological complications, including cardiac perforation and pulmonary embolism. In some cases, catheter migration is asymptomatic and discovered incidentally. When catheter fragments migrate, removal via a percutaneous transvenous approach is preferred to avoid complications. However, in asymptomatic patients, observation may be a suitable alternative if the fragment adheres to the vessel. We report the case of a 25-year-old patient under surveillance for metastatic medullary thyroid carcinoma, whose catheter migrated to the pulmonary artery and remained complication-free for 6 years. This case highlights the importance of routinely checking for pinch-off syndrome whenever a central catheter is placed, and it suggests that removal of migrated catheter fragments may not be necessary in asymptomatic patients.

## Introduction

Implantable catheter ports have become an essential tool in the management of patients receiving chemotherapy, allowing an easy venous access and safe handling of various medications.^1^ By providing long-term intravenous access, this device has significantly improved patients’ quality of life and facilitated the care they receive. It is placed in the operating room by a surgeon, anaesthesiologist, or radiologist.^1^ It can be inserted in the jugular or subclavian vein, with its distal end located in the superior vena cava. However, its placement is not without risk, with thromboembolic events, infections, and catheter dysfunction as primary concerns. Among the most feared complications is the pinch-off syndrome (POS), defined as an intermittent compression of the catheter between the clavicle and the first rib. It can be asymptomatic or lead to an obstruction with an inability to inject saline solutions or aspire. In rare cases, the catheter can break (fracture) and embolise in the cardiac cavity or pulmonary arteries.^2^ We report the case of a 25-year-old patient under follow-up for metastatic medullary thyroid carcinoma who had an implantable port catheter placed via the subclavian vein. A follow-up CT scan revealed a catheter fracture due to POS.

## Case presentation

**Figure 1.**
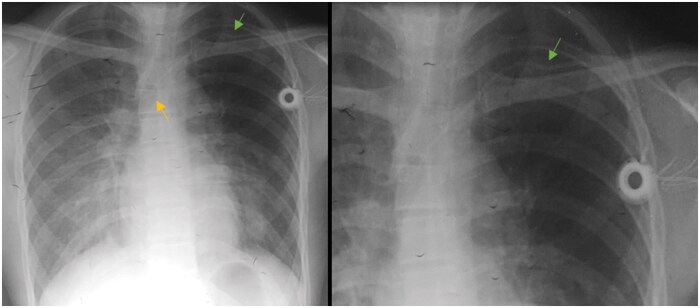
(A) Chest X-ray performed to control the position of the central catheter in the left subclavian vein with the tip ending in the superior vena cava (yellow arrow). (B) Catheter was compressed at thoracic inlet between first rib and clavicle (green arrow), consistent with “pinch-off sign”, grade 1.

A 25-year-old man with no medical history, diagnosed with medullary thyroid carcinoma metastatic to the lungs and lymph nodes, underwent a left-sided implantable port to initiate chemotherapy after surgery. The catheter’s position was confirmed by a control X-ray performed afterward (Figure 1A) with its tip ending in the superior vena cava. The catheter was accessed, the drugs were administered without a problem, and he finished his chemotherapy with a good response and was then under regular monitoring.

**Figure 2.**
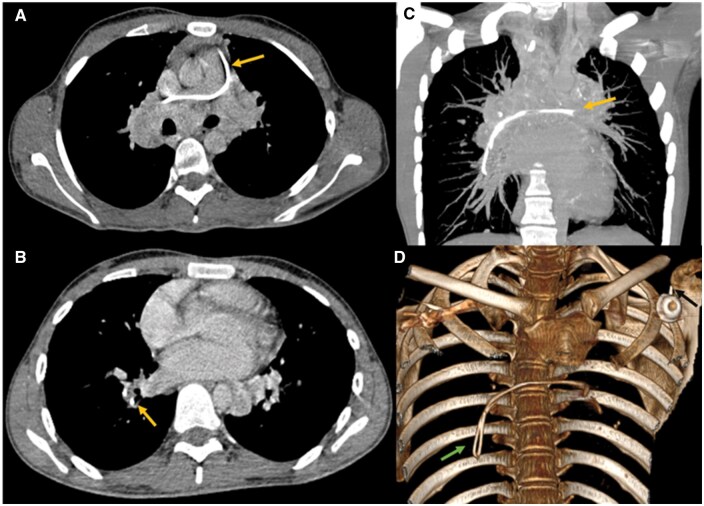
(A) Axial chest CT scan mediastinal window showing the fragmented catheter in the pulmonary trunk extending to the right pulmonary artery. (B) Axial chest CT scan mediastinal window showing, the fragmented catheter the inferior right lobar artery (arrow). (C) Chest CT scan with maximum intensity projection reconstruction in coronal view showing the fragmented catheter extending from the pulmonary trunk to the inferior right lobar artery (arrow). (D) 3D reconstruction showing the external chamber (black arrow) and the fragmented catheter (green arrow).

Two years later, during a routine CT-scan, a detached fragment of the catheter was noted, with material observed in the main trunk of the pulmonary artery, extending to its right branch and the ipsilateral lower lobar artery (Figure 2). After further questioning, he mentioned a fall during a football match, landing on his left shoulder 6 months before the CT scan. The patient experienced no respiratory discomfort or chest pain following the event, explaining why he didn’t seek medical advice. Physical examination was unremarkable. The electrocardiogram and all blood investigations including cardiac enzymes, were normal. Although it was not noted at the time, retrospective review of the first control X-ray showed the catheter compressed between the first rib and the clavicle at the costoclavicular junction (Figure 1B). The diagnosis of catheter fracture due to pinch-off syndrome was established.

The patient was referred for removal of the fractured catheter via a percutaneous endovascular approach, under fluoroscopic guidance. Angiography was performed, the guide wire showed no floatation on fluoroscopy and appeared to adhere to the vascular wall. Therefore, conservative management was decided. The patient is currently under close surveillance, with no symptoms reported over a 6-year follow-up period.

## Discussion

Totally implantable central venous access devices are used in patients with chronic conditions that require long-term venous access for extended therapies. Due to their low risk of infection and extravasation, they are widely used in oncology, allowing the administration of vascular noxious medications such as chemotherapy and parenteral nutrition.^3^^,^^4^

The system consists of a central catheter, most often inserted into the subclavian or internal jugular vein to the superior vena cava, and tunnelled subcutaneously. This catheter is attached to an external subcutaneous port. Placement is generally performed in the operating room under fluoroscopic or ultrasound guidance, with strict aseptic conditions.^2^^,^^4^

After implantation, a chest X-ray is mandatory to verify the position of the venous catheter and its ending in the superior vena cava, identify any immediate complications, such as pneumothorax, but also to look for the “pinch-off sign”, as defined by Hinke and al. as 4 grades:^5^

- grade 0: no narrowing in the catheter’s course/normal.
- grade 1: deviation of the catheter with no luminal narrowing.
- grade 2: luminal narrowing as the catheter passes under the clavicle (pinch-off sign).
- grade 3: transection of the catheter between the clavicle and the 1st rib with embolization of the distal catheter.

Indeed, the placement of this type of catheter is not without risk, estimated at 15%-27%. We distinguish between early complications (malposition, arrhythmia, perforation and bleeding, air embolism, pneumothorax) occurring <30 days after complication and late complications that occurs > 30 days, such as infection, thrombosis or venous stenosis, and especially catheter dysfunction, including malposition, POS, catheter fracture and migration.^3^^,^^4^

Catheter dysfunction are most often suspected if there is an inability to inject saline solutions, subcutaneous extravasation, prolonged infusion time, subcutaneous swelling, cervical or back pain, as well as an inability to aspirate from the port or obtain venous return.^3^

In our case, the patient had a POS, defined as intermittent compression of the catheter between the clavicle and the first rib. This complication is specific to the subclavian vein approach.^2^^,^^4^ In the short term, it leads to dysfunction and decreased flow through the catheter, or difficulty with aspiration and extravasation. In the long term, following an increase in intrathoracic pressure (due to coughing, sneezing, weight lifting, position changes, or falls), the forces exerted will be responsible for catheter fracture or disconnection, and its migration to the inferior vena cava, the right cardiac cavity, and in rare cases, to the pulmonary arteries.^4^

The migration of the catheter can be responsible for neurological and cardiovascular complications, such as tachycardia or arrhythmia, cardiac perforation, pulmonary pseudoaneurysms, and infections.^6^ It also contributes to flow stagnation in the vessel and clot formation, increasing the risk of embolism and underlying pulmonary infarction.^3^^,^^6^ In some cases, it may be asymptomatic, with incidental discovery during routine exams,^6^^,^^7^ as in our case.

It is important to note that POS can occur after catheter insertion or later, hence the importance of close monitoring of the device.^2^

Most catheter fragments are removed soon after discovery to avoid complications.^7^ In cases of catheter disconnection or fragmentation with embolization, the percutaneous transvenous approach (via the femoral vein) is the method of choice; thoracotomy or other surgical interventions would seriously increase morbidity and mortality.^2^^,^^3^ With a guiding catheter, a goose-neck loop is maneuvered towards the fragment to catch the tip. Once the loop is tightened around the catheter (fragment), it can be safely retrieved.

In patients where catheter fragmentation is diagnosed late, the formation of a fibrin sheath around the catheter, with adherence to the vessel or endocardium, may prevent extraction.^3^ Indeed, two cases have been reported in the literature of a catheter migrating to the pulmonary artery without clinical sequelae or risk of further migration, one for 14 years,^8^ while the other was removed after 11 years due to a risk of endocarditis, with no notable adhesions.^9^ Such was the case for our patient, whose diagnosis was made 6 months after the trauma. Those cases further illustrate that removal of a catheter fragment is not absolutely necessary in asymptomatic patients.^7^ Our patient did not experience any complications from the retained catheter fragment for 6 years.

Finally, to prevent POS, it would be advisable to use the jugular vein approach or to insert the catheter as laterally as possible in the subclavian vein.^2^^,^^10^

## Conclusion

Central venous catheters are useful for patients who require long-term treatment, but complications may arise as a result of this procedure. In certain cases, POS may go unnoticed, leading to catheter fracture and migration, discovered during routine exams. Although catheter fragments are usually removed to prevent complications, some cases have shown that asymptomatic patients can safely retain fragments for years. To avoid complications such as pinch-off syndrome, it is advisable to use the jugular vein approach or position the catheter laterally in the subclavian vein.

## Learning points

- Central venous catheters are widely used in oncology and can be placed in the jugular or subclavian vein.
- Complications of central venous catheters include both short-term and long-term ones, mainly catheter dysfunction.
- A control X-ray is necessary after placement to check the catheter’s position, assess for complications, and look for the pinch-off sign, a warning of pinch-off syndrome. Pinch-off syndrome can lead to catheter fracture and migration into the vena cava, cardiac cavity, or pulmonary artery.
- Treatment for catheter migration involves removing the embolized fragment with an endovascular approach. However, in some cases where the patient remains asymptomatic and the catheter is adherent to the vascular wall, abstention can be an option.
